# Intelligent prediction and optimization of nanofluid-assisted turning using hybrid generative deep learning

**DOI:** 10.1038/s41598-026-50277-9

**Published:** 2026-04-29

**Authors:** Neelesh Kumar Sahu, Roja Abraham Raju, Ruchi Patel, Ankur Jaiswal

**Affiliations:** 1 Department of Mechanical Engineering, Marwadi University, Rajkot, 360003 Gujarat India; 2 Department of Mechanical Engineering, Mar Athanasius College of Engineering, Kothamangalam, 686666 Kerala India; 3 Department of Computer Engineering, Marwadi University, Rajkot, 360003 Gujarat India; 4 Manipal Institute of Technology, Manipal Academy of Higher Education, Manipal, India

**Keywords:** Nano fluid, Cutting force, Surface roughness, RSM, GAN- ANN, Explainable-AI hybrid RSM-ANN desirability-based optimization, Engineering, Mathematics and computing

## Abstract

Nanofluid-assisted machining is a promising sustainable alternative to the conventional cutting fluids; however, the correct prediction and optimization of the latter process is still difficult due to nonlinearities inherent to it and the lack of experimental data. Hence, the current study presents an innovative hybrid generative deep-learning model that synergistically integrates Response Surface Methodology (RSM), Artificial Neural Networks (ANN), and a Generative Adversarial Network (GAN) to predict intelligently and optimize multi-fold in the nanofluid-assisted turning of EN31 steel. It has been experimentally shown that MWCNT based gravity fed nanofluid decreases the surface roughness by 12.83% and cutting force components by up to 23.4% when compared to traditional coolant. The RSM model has a moderate predictive power (R^2^ ≈ 0.82–0.88) and ANN model outperforms the RSM model (R^2^ ≈ 0.95–0.97). Under the limited data condition, the GAN-ANN hybrid has higher predictive robustness with R^2^ ≈ 0.97–0.99 and lowest mean squared error (1.49 for roughness and 7.89 for cutting forces) than any of the other models tested. Using a hybrid ANN-RSM strategy based on desirability, best machining parameters predict a cutting force of about 205 N, a surface roughness of 0.45 μm and a maximum rate of material removal of 16.6 m^3^/min, which are later confirmed in follow-up experiments with an error of less than 5%. Therefore, the suggested framework will provide a scalable, interpretable and environmentally friendly decision support system to intelligent machining applications.

## Introduction

The velocity of cutting is limited when machining is done without coolant because of the increased temperature levels in the tool-work contact point due relative motion and friction^1^. Threshold value of cutting tool wear reach fast when subjected to high temperatures, which reduces their durability and tool life. In order to overcome these obstacles in high-speed material removal, it is crucial to use the right coolant^2^. Reducing friction with good wettability, high thermal conductivity, and chip removal from the cutting zone are the main benefits of using traditional cutting fluids. Although these fluids have been useful, there are worries about their misuse and the risks they represent to the atmosphere and human well-being^3^. Simultaneously, the predominant use of conventional coolant as flooded manner increases production expenses in the machining sector, encouragement a transition to more efficient alternatives^4^. The optimization of performance and firmness of heat exchangers is intrinsically linked to the heat transfer competence of coolant. The less heat carrying rate of these fluids continues to be a significant barrier. The development of innovative nanofluids utilizing solid elements as additives is a viable approach to address the challenge^5^.

In machining, power consumption, surface roughness and tool life are the major factors which affect the machining cost. ‘Cutting force’ in removing material is straight influencing power consumption. If ‘cutting force’ is reduced, cost of machining could be greatly reduced as the energy required for machining is reduced. Machining parameters, work piece material, tool geometry, condition of machine tool, vibration and type of cutting fluid used are the factors influencing cutting forces^6^. Roughness of machined surface is a significant factor because the irregularities on the surface may nucleate cracks, causes corrosion there by reducing the fatigue life of a product^7,8^. Another important parameter to be considered while machining is tool life. Tool life is crucial role in reducing machining cost^9^. Many researches were carried out for finding an optimal cutting parameter for machining different materials for maximizing life of tool & MRR and minimizing ‘cutting forces’ & ‘surface roughness’^10–12^. Cutting fluids used in machining improves tool life, surface finish of work piece and reduces machining forces, thereby decreasing power requirement and increasing life of tool^13–15^. Nanoparticles have the potential to change sliding friction into a hybrid of the two types of friction by creating a rolling action at the mating surfaces. Incorporating nanofluids also improves the lubrication and thermal stability^16^. To top it all off, the presence of nanoparticles in the carrying liquid may have helped advance the mixing flow and offered better thermal conductivity^17^.

Recently researches put forward the use of efficient nano fluids as an alternative for cutting fluid in machining^18,19^. Das et al. (2019)^20^ studied several nanofluids characterized by the hard turning of AISI 4340 steel. Typically, three groups of nanofluids were produced, namely Al_2_O_3_, CuO, and Fe2O3 and overall, the CuO nanofluid performed the best, followed next by Fe2O3 and Al2O3. In later studies, Das et al. (2021)^21^ studied fluid properties of parameters such as ‘thermal conductivity’, ‘viscosity, ‘surface tension’, and ‘contact angle’. Similarly, Andhare and Roja (2016)^22^ looks at the effectiveness of multiwalled carbon nanotubes (MWCNTs) suspended in distilled water with the aid of sodium dodecyl sulfate (SDS) as a suspending agent to enhance the properties of traditional cutting fluids. The following study systematically evaluated a variety of concentrations of MWCNTs and looked in detail at the thermal conductivity, pH, viscosity, and wettability. There results claim that prepared nanofluid with MWCNT, found enhance in coolant properties. Further in their work, Sahu et al. (2018)^23^ investigated the performance of developed MWCNT based nanofluid coolant during turning of Ti–6Al–4 V. The machined responses measured with MWCNTs-based nanofluid provide more desirable outcomes such as a 34% drop in tool wear, on average 28% drop in cutting forces and on average 7% drop in ‘surface roughness’ in comparison to conventional fluids as coolant.

In other work with MWCNT based nanofluid, Hegab et al. (2018)^24^ investigates the use of minimum quantity lubrication (MQL) with MWCNTs dispersed in vegetable oil to improve the machinability of the titanium alloy Ti–6Al–4 V. The study found that 2 wt% of MWCNTs reduced power by 11.5% and flank wear by 45% compared to conventional MQL (without additives). Park et al.^25^ investigated the effects of adding graphene (xGnP) to an oil-based lubricant formulation. They noted improved cutting surface wettability and encouraging results regarding surface friction. The most effective cutting capability was seen at a loading concentration of 0.1 wt% graphene. When comparing tool life and cutting zone temperature, MWCNT showed superior efficiency. From above literature it is cleared that nanofluids are showing better results in machining performance compared to conventional fluids. The development of a tribo-film, the sliding-rolling mechanism, and the restorative capabilities of nanoparticles are the key elements that enhance surface finish and diminish ‘cutting temperature’, ‘tool wear’, and ‘cutting force’ in machining with nanofluids and hybrid nanofluids^26^.

The efficacy of optimization methods for machining processes under nanofluid conditions has been demonstrated in several published research articles. To optimize the turning settings of an aluminium metal matrix nanocomposite, Chakma et al. (2022)^27^ employed the Taguchi orthogonal array design. When using carbon nanotube nanofluid, the investigators found that high speed of cutting with modest insert-feed/rev greatly improved surface quality. Liew et al. (2017)^28^ optimized the turning of D2 steel using the Taguchi-RSM integration approach for multiple responses. Cutting at 144.58 m/min with an insert advancement/rev of 0.14 mm/rev and using carbon nanofiber nano fluid as a coolant yields the best tool wear and surface finish, according to the trial data. Along those lines, Barewar et al. (2021)^29^ used the Taguchi grey relational analysis (TGRA) to study the impact of velocity of cutting, feed rate, and machining environment (Ag/ZnO hybrid nanofluid-MQL, dry, and MQL) on cutting temperature and roughness of machined surface during Inconel 718 milling. They found that a cutting environment with an Ag/ZnO hybrid nanofluid-MQL, speed of cutting of 30 mm/min, and insert-feed/rev of 0.036 mm/tooth could achieve multi-response-optimized machining performance. The value of the parameter mixture for forecasting the optimal micro drilling force and torque deviated from the experimental values by only 0.44% and 1.24%, respectively, according to Huang and Chen (2020)^30^, who utilized ANN and TGRA to create a highly accurate micro drilling prediction model. From the above literature predicting the optimal parameters to enhance material removal effectiveness beneath nanofluid and hybrid nanofluid cutting environments is made possible by multi-response optimization employing various optimisation approaches including Taguchi, RSM, and ANN.

The recent studies on machining optimisation have progressively used the advanced computational tools in terms of improving the performance and accuracy of the model. As an illustration, neural network-based strategies have been effectively used to model and optimise complex machining processes and have shown the ability to strongly predict under different conditions^31^. Equally, genetic algorithms and evolutionary strategies are evolutionary computation approaches that have originate extensive submission to find optimal machining parameters in multi-objective sceneries^32,33^. These works indicate the usefulness of hybrid and intelligent-based optimisation schemes, which justifies our intended multi-source contextual and behavioural fusion model. Besides machining with nanofluids, other sustainable machining processes, including cryogenic-assisted turning and novel electrical discharge machining (EDM) have been explored to increase the machinability of high-temperature superalloys^34,35^. Similarly, EDM-based processes with environmental concerns have been used to machine Nimonic C -263 and Haynes 25 superalloys, such as the use of additively manufactured CuCr -Zr electrodes to make precision small holes.

However, most of the literature is focused on the experimental performance assessment, and relatively small datasets development of intelligent data-driven models, and generative augmentation methods are relatively underdeveloped.

Based on the overall literature review it is found that MWCNT nanoparticles are having higher thermal conductivity, and good lubricating properties compared to other nanoparticles. Despite improving machining performance relative to traditional fluids, the stability of nanofluids remains a challenge in actual use. Massive particle clustering and agglomeration had the greatest impact on nanofluid stability^36^. In numerous studies, researchers have employed MQL or cryogenic techniques combined with nanoparticles, yielding superior outcomes. However, this will further elevate machining costs because to additional setup necessities, including pressurized air and a mixing chamber in the case of MQL. Likewise, in a cryogenic arrangement, the storage and movement of liquid CO_2_ or N_2_ will elevate machining expenses.

Although the current research has made extensive progress in the field of nanofluid-assisted machining, most of the studies that have been conducted are limited to experimental analysis or application of single-model prediction framework. However, machining processes are nonlinear and data-intensive and expensive to test, the combination of which leads to small datasets that limits the trustworthiness of isolated machine-learning models. Scientifically speaking, there is still an urgent need to integrate physics-conscious experimental modelling with data-driven generative intelligence that would enhance predictive resilience in the circumstances of data scarcity.

Industrial wise, the manufacturing industries require a sustainable, cost efficient, and interpretable decision-support systems that are capable of minimising cutting forces, improving surface quality and maximising productivity and, at the same time, minimising coolant usage and energy consumption. Traditional MQL and cryogenics require a costly infrastructure and hence can only be used in small and middle scale businesses. As a result, a scalable, cost-effective, and environmentally friendly solution to intelligent machining is the creation of a gravity-fed nanofluid system combined with a hybrid GAN-ANN predictive and optimisation system.

The combination of nanofluid-assisted machining with a hybrid Generative Adversarial Network–Artificial Neural Network (GAN–ANN) predictive framework and multi-objective optimization for sustainable turning performance enhancement, adds the novelty in the present work.

The significant contributions and the new things of this work can be summarized as the following points:

1. A gravity-fed multi-wall carbon nanotube/based nanofluid system is combined with intelligent predictive model to obviate expensive pressurised MQL or cryogenic systems and thus offers significant industrial scalability.
2. The appearance of hybrid GAN-ANN framework responses the problem of experimental data scarcity in machining, making it possible to augment synthetic data physics-consistently to enhance predictive robustness.
3. The predictive equations depend on comprehensive statistical validation of response- surface methodology (RSM) models, including analysis of variance, 95 probability interval, residual diagnostics and multicollinearity test.
4. Explainable artificial intelligence (SHAP) is used to interpret the effect of machining parameters to address the black-box nature of deep learning models.
5. The experimental validation of a hybrid ANN-RSM multi-objective optimisation framework, with the prediction errors of less than 5%, makes it industrial feasible.

**Fig. 1:**
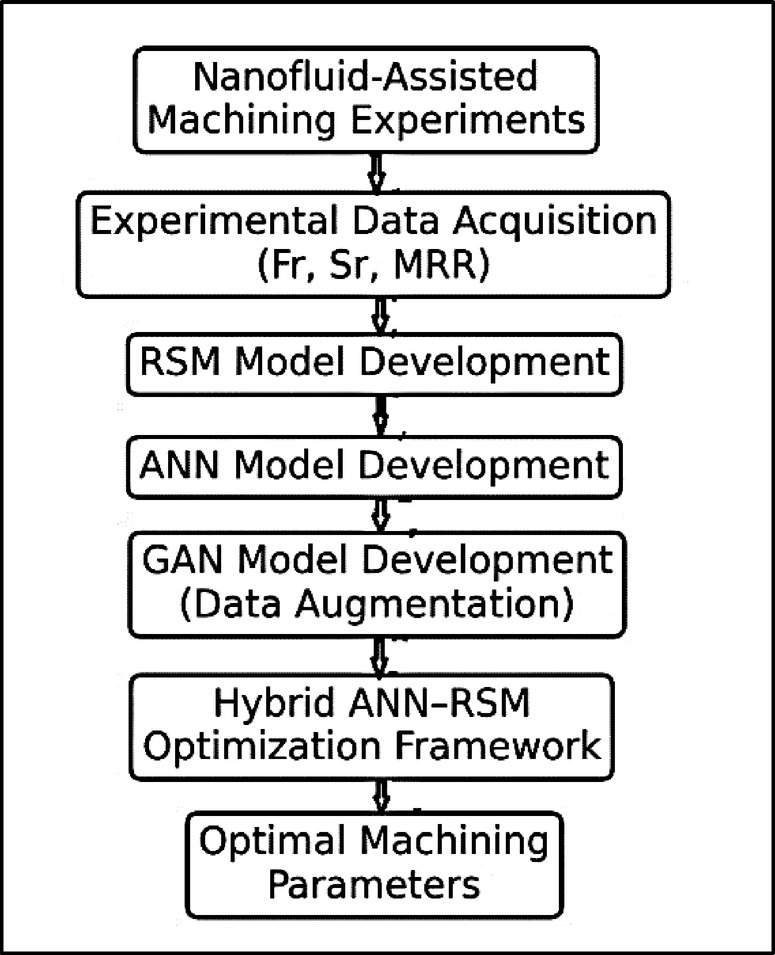
The proposed framework integrating nanofluid-assisted machining experiments, RSM modeling, ANN and GAN-based learning, and a hybrid ANN–RSM optimization framework for intelligent machining performance optimization.

Previous studies focus solely on experimental evaluation or use single-model prediction techniques, whereas this work introduces a three-stage methodological pipeline that combines experimental validation, intelligent modelling, and evolutionary optimization. This work exclusively demonstrates the preparation and deployment of a gravity-fed MWCNT-based nanofluid (reduces the coolant consumption to 1 L/hour), and eliminating the necessity for pressurized systems, thus contributing to eco-friendly machining. The GAN–ANN model also ingeniously resolves the problem of less experimental datasets by generating genuine synthetic data to make predictions about ‘cutting force’ and ‘surface roughness’ more accurate (R² ≈ 0.97). In an attempt to be more transparent about the ANN and GAN-ANN model, SHapley additive exPlanations (SHAP) were used to estimate how much of the contribution of individual machining parameters. The work presented in this paper is divided into three stages as shown in Fig. 1.

## Experimentation

### Preparation of nano fluid

MWCNT’s were dispersed in single distilled water for producing nanofluid. Since MWCNTs will not directly disperse in water, Sodium dodecyl sulphate (SDS) is used as a surfactant. Volume fraction of both nano fluid and surfactant is very important in obtaining desired properties in nanofluid. So, from our previous research we have selected 0.2 volume % MWCNT, with 0.5 volume % of SDS shows the most favorable properties of a cutting fluid^22^. As the percentage of SDS increases, thermal conductivity is negatively affected and also SDS is a forming agent. Blasocut 4000 strong oil is used to make conventional cutting fluid. Concentration of oil in conventional cutting fluid were taken as 20% a per the catalogue. Table 1 gives the key features of both fluids and nanoparticle.

**Table 1:** Features of nanofluid and conventional cutting fluid.

Particulars	Features
MWCNT	OD: 10–20 nm, Length 1–2 μm, Purity: 95% Density: 2 gm/cm3
SDS	Density: 1.01 gm/cm3
Cutting oil (Blaso Cut 4000 Strong)	Mineral Oil 45%, chlorine 6% Density 0.99
Conventional Cutting fluid	20% cutting oil + 80% Water
Nano fluid	MWCNT: 0.2 Vol% SDS: 0.5 Vol% Remaining: Distilled water

### Properties of nano fluid

Andhare and Roja (2016)^22^ have evaluated MWCNT based nano fluids and determined their properties like thermal conductivity, contact angle, viscosity and pH. It was found that nano fluid has favorable properties such as cutting fluids. The most important properties out of the above are thermal conductivity (to carry heat) and contact angle (better wettability - to reduce friction). These properties are therefore confirmed once again. The fluid has a thermal conductivity of 0.751 W/m-K at 28 °C, which was measured using KD 2 Pro thermal properties analyser. Also, the contact angle of a sessile droplet of nanofluid is 24º, measured using SEO Phoenix 300 contact angle analyzer. Thermal conductivity of distilled water is 0.588 W/m.K and contact angle is 70º. Hence there is a good increase in thermal conductivity and decrease in contact angle of nano fluid in comparison with water. Hence, nanofluid with higher thermal conductivity and high wettability makes it an alternative for cutting fluids in machining.

### Turning procedure

**Fig. 2:**
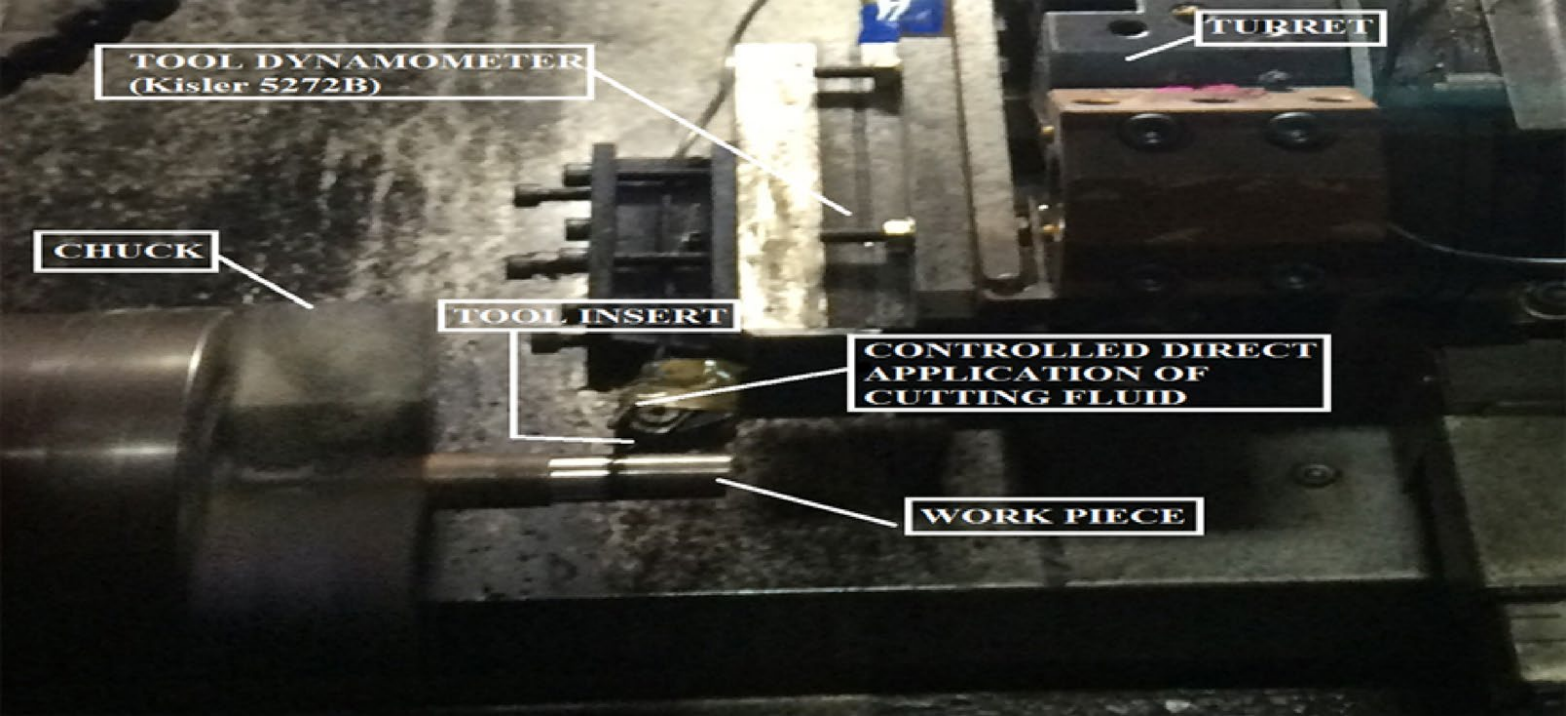
Experimental setup for turning with gravity feed for coolant.

Turning was executed using a CNC lathe with a spindle power of 5.5 kW. TNMG 160408CQ inserts, coated with CVD on carbide, were utilised for turning operations. Cutting procedures were performed on EN31 workpieces. Figure 2 illustrates the machining configuration and the application of cutting fluid to the workpiece.

Table 2 shows the specification and composition of work piece material being used. Turning was performed in three different conditions – dry, with conventional fluid and with nanofluid. Keeping two parameters constant and varying the third parameter, the effect on machining forces (*F*_*x*_, *F*_*y*_, *F*_*z*_) and surface roughness were studied under three machining conditions.

To measure ‘cutting forces’, the Data Acquisition System and LabVIEW software from National Instrument were utilised. The “Surface Roughness Tester”, manufactured by Mitutoyo, was used to measure the surface’s roughness. You can see the main characteristics of the experimental equipment in Table 3.

**Table 2:** Work piece specification & composition.

Workpiece	EN 31 (ϕ25mm X 110 mm)		
Composition			
C	0.88%	Cr	0.44%
Si	0.29%	Mo	0.04%
S	0.04%	Ni	0.18%
M	1.74%	Al	0.02%
P	0.05%	Cu	0.06%

**Table 3:** Specification of equipment’s used for the study.

Equipment used	
Machine tool- MAXTURN +	Siemens control, spindle power 5.5 KW; Accuracy in axes positioning 0.01 mm, Resolution- 0.001 mm
Surface Analyzer	Surftest SJ-410 Mitutoyo, Japan, Resolution-0.001 μm
Tool Dynamometer- Kistler 9257BA	-5 to 10KN, Top plate 100 to 170 mm Sensitivity − 10.0mV/N

## Assessment of nanofluid in turning operation

The main objective behind this study is to confirm whether nanofluid could be an alternate to conventional cutting fluid used while turning. To assess the impact of nanofluid on machining, the nanofluid is examined under varying speeds, feeds, and turning depths. The efficiency of nanofluid could be analyzed by measuring surface roughness and ‘cutting forces. An efficient cutting fluid reduces surface roughness and machining force significantly. All experiments were repeated 3 times and the average is considered. The results of the experiments that were conducted to examine the performance of nanofluids are displayed in Table 4.

**Table 4:** Experimental Investigation of nanofluid in turning operations.

Parameter	Symbol	Levels	Effect on ‘surface roughness’ (S_*r*_)	Inference on ‘cutting forces’ (F_x_, F_y_, F_z_ )	Overall observation
Cutting speed (RPM)	*C*_*s*_	650–2500	12.83% decrease in surface roughness was obtained while using nano fluid when compared with conventional cutting fluid.	An average of 10.64% decrease in ‘cutting force’ (*F*_*x*_), 23.4% decrease in Thrust force (*F*_*y*_) and 14.24% decrease in feed force (*F*_*z*_)	Because of its short contact angle and extremely strong ‘thermal conductivity’, nano fluid outperforms traditional Blaso-cut cutting fluid.
Insert-feed per revolution (mm/rev)	*I*_*r*_	0.016–0.184			
Turning depth (mm)	*D*_*c*_	0.5–1.84			
Coolant Strategies		Dry, Conventional coolant, Nanofluids			

### Surface roughness

When nanofluid is used, the roughness values are still lower than those while using conventional fluid. In case of nanofluid, wetting area per volume of liquid at the workpiece-tool surface improves as ‘surface roughness’ decreases. Recognizably, raising the feed rate raises surface roughness while raising the speed of cutting lessens it. This is attained with better wettability of the nanofluid which enables it to act as a better lubricant. During high-speed operation due to very high temperature, surface roughness can increase but while using nanofluid better cooling of cutting zone, heat is easily removed from the cutting zone due to higher thermal conductivity of the nanofluid, thereby reducing surface roughness.

### Machining forces

The machining force is an essential metric for measuring the power used and energy wasted by the machine tool when turning. But there are a lot of factors that affect the main machining force during turning. These include things like the wear of the cutting insert, the thermo-mechanical properties of the workpiece and tool material, and the unique cooling environment. Machining forces are mainly ‘cutting force’ (*F*_*x*_), thrust force (*F*_*y*_) and Feed Force (*F*_*z*_). These 3 components of forces were retrieved using a dynamometer which is fixed on the turret of the machine with the help of a fixture. This may arise from the augmented chip flow at elevated feed rates, which reduces the coefficient of friction at the tool–workpiece interface^24^. Another factor contributing to this phenomena is the lowering of chip thickness with an increase in velocity of cutting. Indeed, at a reduced velocity of cutting, the increase in temperature was diminished due to decreased plastic deformation and shearing.

With reference to previous work^37^ it is clear that there is a signification effect of feed and turning dpeth on ‘cutting force’ where as speed remain insignificant. This condition is same for 3 components of forces. In all experiments in determining force components, it was clearly seen that ‘cutting force’ has been reduced while using Nano fluid. The primary reason for the diminished values of the main cutting force in nanofluid circumstances is the presence of MWCNT nanoparticles, which may have created a ball-bearing effect at the tool-chip interface, hence reducing the total coefficient of friction^38^.

This is mainly due to the wettability and the capillary penetration of the Nano fluid. Nano fluid properly wet the chip and tool interphase. One third of total cutting force in machining is liberated as heat in order to overcoming friction between tool and the work piece. Favorable properties of nanofluid reduce friction and temperature rise, which ultimately lead to reduced cutting force as compared with dry machining and machining using conventional cutting fluid. Research has shown that reducing the size of nanoparticles and increasing their concentration in the base cutting fluid significantly improves thermal conduction while cutting. In nanofluid condition, reduced friction coefficient and improved heat conductivity may diminish cutting forces. The enhancement in heat conductivity may yield improved outcomes regarding tool longevity, temperature, surface finish, and cutting forces. Nanofluid is a better alternative for conventional cutting fluid in machining as it reduces quantity of fluid consumption and reduces air pollution.

## Development of prediction models in nanofluid conditions

Based on assessment on nanofluid in turning operation, further experiments are performed with nanofluid as a cutting fluid while turning. The cutting parameter ranges (cutting speed, insert-feed per revolution, and Turning Depth) are identical to those utilized for assessment of nanofluids. These ranges are evaluated in relation to the cutting tool catalogue for the workpiece material and preceding literature as shown in Table 5. The initial stage in developing prediction models is to generate data for the input and output parameters. Consequently, data is generated through tests conducted in a systematic sequence, accomplished via experimental design. The Response Surface Method (RSM) was employed for experimental design, comprising 20 experimental sets utilizing Central Composite Design (CCD), as illustrated in Table 6. The CCD has five operational variables, including axial (α = ±1.681), factorial (± 1), and center points (0). When a design has k factors, the axial points are situated at a distance of α = 2^(k/4) from the design center. In the present work, for the generation of prediction model for ‘cutting forces’, the resultant value of the all the three components of machining force (*F*_*x*_, *F*_*y*_, *F*_*z*_) using Eq. (2) is taken into consideration. The ‘surface roughness’ values, MRR, and ‘cutting forces’ findings are summarized in Table 6.

**Table 5:** Level of turning parameters as per CCD.

Level	Cutting speed (m/min) C_s_	Cutting speed (RPM)	Insert-feed per revolution (mm/rev) I_*r*_	Turning depth (mm) D_c_
Axial α = -1.681	51.7	659	0.016	0.16
Factorial − 1	78.5	1000	0.05	0.5
Center 0	117.75	1500	0.1	1
Factorial + 1	157	2000	0.15	1.5
Axial α = +1.681	183.77	2341	0.19	1.84

**Table 6:** List of experiments based on central composite design used in RSM and Machining response observed after conducting each experiment.

Cutting speed (RPM)	Cutting speed (m/min) C_s_	Insert-feed per revolution (mm/rev) I_*r*_	Turning depth (mm) D_c_	Resultant cutting force F_*r*_ (*N*)	Surface Roughness S_*r*_ (µm)	MRR (m^3^/min)
1000	78.5	0.15	0.5	158.39	2.85	5.8
2000	157	0.15	1.5	287.9	0.676	16.6
1500	117.75	0.19	1	248.17	0.99	13.9
1500	117.75	0.016	1	156.07	2.604	1.2
1000	78.5	0.15	1.5	262.7	0.48	16.6
1500	117.75	0.1	1.84	235.54	0.72	13.4
2000	157	0.05	0.5	132.39	0.36	1.9
2000	157	0.05	1.5	204.29	1.87	5.5
1000	78.5	0.05	0.5	81.05	2.87	1.9
1500	117.75	0.1	1	177.12	0.49	7.5
659	51.7	0.1	1	157.23	2.446	7.5
1000	78.5	0.05	1.5	140.2	0.703	5.5
1500	117.75	0.1	1	173.9	0.495	7.5
2341	183.77	0.1	1	185.24	0.433	7.5
2000	157	0.15	0.5	165.83	1.16	5.8
1500	117.75	0.1	1	164.92	0.47	7.5
1500	117.75	0.1	1	179.72	0.45	7.5
1500	117.75	0.1	0.16	50.54	2.25	1.2
1500	117.75	0.1	1	176.79	0.485	7.5
1500	117.75	0.1	1	167.93	0.48	7.5

The machining responses are measured during and after each experiment such as cutting forces measured turning operation and surface roughness are measured after the experiment. However, MRR in m^3^/min is calculated by the empirical formula as shown in Eq. (1).

1 $$\:MRR={C}_{s}\times\:{I}_{r}\times\:{D}_{c}$$

Where *C*_*s*_ = cutting speed in m/min; *I*_*r*_ = insert-feed/rev in mm/rev; *D*_*c*_ = turning depth in mm.

In the present work, for the generation of prediction model for ‘cutting forces’, the resultant value of the all the three components of machining force (*F*_*x*_, *F*_*y*_, *F*_*z*_) using Eq. (2) is taken into consideration. The ‘surface roughness’ values, MRR, and ‘cutting forces’ findings are summarized in Table 6.

2 $$\:{F}_{r}=\:\sqrt{{{F}_{x}}^{2}+{{F}_{y}}^{2}+{{F}_{z}}^{2}}$$

### Prediction model for surface roughness and cutting forces based on RSM methodology

RSM is essential to experimental design. It provides important mathematical and statistical tools for modelling and analysing situations with multiple components influencing a response variable. In many industrial situations, accurate modelling is possible using a second-degree polynomial^39^. This technique produces the second-order model below Eq. 3.

3 $$\:{\boldsymbol{M}}_{\boldsymbol{r}}={\boldsymbol{m}}_{0}+\sum\:_{\boldsymbol{i}=1}^{\boldsymbol{n}}{\boldsymbol{m}}_{\boldsymbol{i}}{\boldsymbol{y}}_{\boldsymbol{i}}+\:\sum\:_{\boldsymbol{i}=1}^{\boldsymbol{n}}{\boldsymbol{m}}_{\boldsymbol{i}\boldsymbol{i}}{\boldsymbol{y}}_{\boldsymbol{i}}^{2}+{\sum\:}_{\boldsymbol{i}=1}^{\boldsymbol{n}-1}\sum\:_{\boldsymbol{j}=\boldsymbol{i}+1}^{\boldsymbol{n}}{\boldsymbol{m}}_{\boldsymbol{i}\boldsymbol{j}}{\boldsymbol{y}}_{\boldsymbol{i}}{\boldsymbol{y}}_{\boldsymbol{j}}+\boldsymbol{e}$$

The measured response, M_r_, in this study is defined as the product of the regression coefficients (*m*_*i*_), the input components (*y*_*i*_), and the estimated error (*e*). Polynomial approximation uses the least square approach to estimate parameters. Afterwards, response surface analysis is carried out using the accuracy fitted surface. The best way to estimate model parameters is to use an appropriate experimental design while gathering data. In this study, we investigate variables such as cutting forces and surface roughness by means of statistical analysis and model creation. Keep in mind that while an RSM model can be created for MRR as well, we can simply estimate MRR from cutting parameters using the well-established mathematical model in Eq. 1. Thus, while the RSM model for MRR is not created for this study, it is utilized as a response for optimization.

### RSM for predicting surface roughness and cutting forces

For each turning operation on EN31 steel, surface finish measurements were taken. We measured the surface roughness at three different locations on the machining surface and averaged the results to account for measurement uncertainties. Similarly cutting force are measured online during turning operation with cutting force dynamometer mounted on the turret of machine. Force data was visualised and stored in computer using LabVIEW software. Using the RSM, we were able to construct a second-order quadratic model like Eq. (3) as shown in Eq. (4) and Eq. (5). This model considers speed of cutting, insert-feed/rev, and turning depth to be the influencing elements on surface finish, with surface roughness serving as the outcome variable. The developed second-order model’s influential elements were identified by an analysis of variance. In order to remove inefficient components from the model, backward elimination was employed. The significant parameters and the accompanying p-values, t-values and R^2^ are shown in Tables 7 and 8.

4 $$\:{S}_{r}=12.79-0.00709\times\:{C}_{s}-37.41\times\:{I}_{r}-7.46\times\:{D}_{c}+0.000001{C}_{s}^{2}+162.2\times\:{{I}_{r}}^{2}+\:1.2\times\:{D}_{c}^{2\:\:\:\:}+0.002782\times\:{C}_{s}\times\:{D}_{c}$$

5 $$\:{F}_{r}=-42.1+0.070\times\:{C}_{s}-73\times\:{I}_{r}+124.6\times\:{D}_{c}+4524\times\:{{I}_{r}}^{2}-37.2\:\times\:{D}_{c}^{2\:\:\:\:}-0.414\times\:{C}_{s}\times\:{D}_{c}+477\times\:{I}_{r}\times\:{D}_{c}$$

**Table 7:** Analysis of Variance for the surface roughness prediction model.

Source	DF	Adj SS	Adj MS	F-Value	*p*-Value
Model	7	14.3141	2.04488	12.38	0.000
Linear	3	6.3382	2.11274	12.79	0.000
Cutting Speed *C*_*s*_	1	2.8352	2.83521	17.16	0.001
Insert-feed per revolution *I*_*r*_	1	0.7926	0.79255	4.80	0.049
Turning Depth *D*_*c*_	1	2.7104	2.71044	16.40	0.002
*C*_*s*_^2^	1	1.1572	1.15723	7.00	0.021
*I*_*r*_^2^	1	2.4074	2.40744	14.57	0.002
*D*_*c*_^2^	1	1.2924	1.29240	7.82	0.016
*C*_s_ x *D*_*c*_	1	3.8684	3.86837	23.41	0.000
Lack-of-Fit	7	1.9814	0.28306	1061.47	0.000
Error	12	1.9827	0.16523		
Pure Error	5	0.0013	0.00027		
Total	19	16.2969			

**Table 8:** Analysis of variance for the cutting force prediction model.

Source	DF	Adj SS	Adj MS	F-Value	*p*-Value
Model	7	57284.3	8183.5	57.98	0.000
Linear	3	52030.0	17343.3	122.87	0.000
Cutting Speed *C*_*s*_	1	2748.5	2748.5	19.47	0.001
Insert-feed per revolution *I*_*r*_	1	16402.3	16402.3	116.21	0.000
Turning Depth *D*_*c*_	1	32879.2	32879.2	232.94	0.000
*I*_*r*_^*2*^	1	1891.9	1891.9	13.40	0.003
*D*_*c*_^*2*^	1	1252.4	1252.4	8.87	0.012
*C*_*s*_ x *I*_*r*_	1	856.8	856.8	6.07	0.030
*I*_*r*_ x *D*_*c*_	1	1136.0	1136.0	8.05	0.015
Lack-of-Fit	7	1526.4	218.1	6.51	0.028
Error	12	1.9827	0.16523		
Pure Error	5	167.4	33.5		
Total	19	58978.1			

**Fig. 3:**
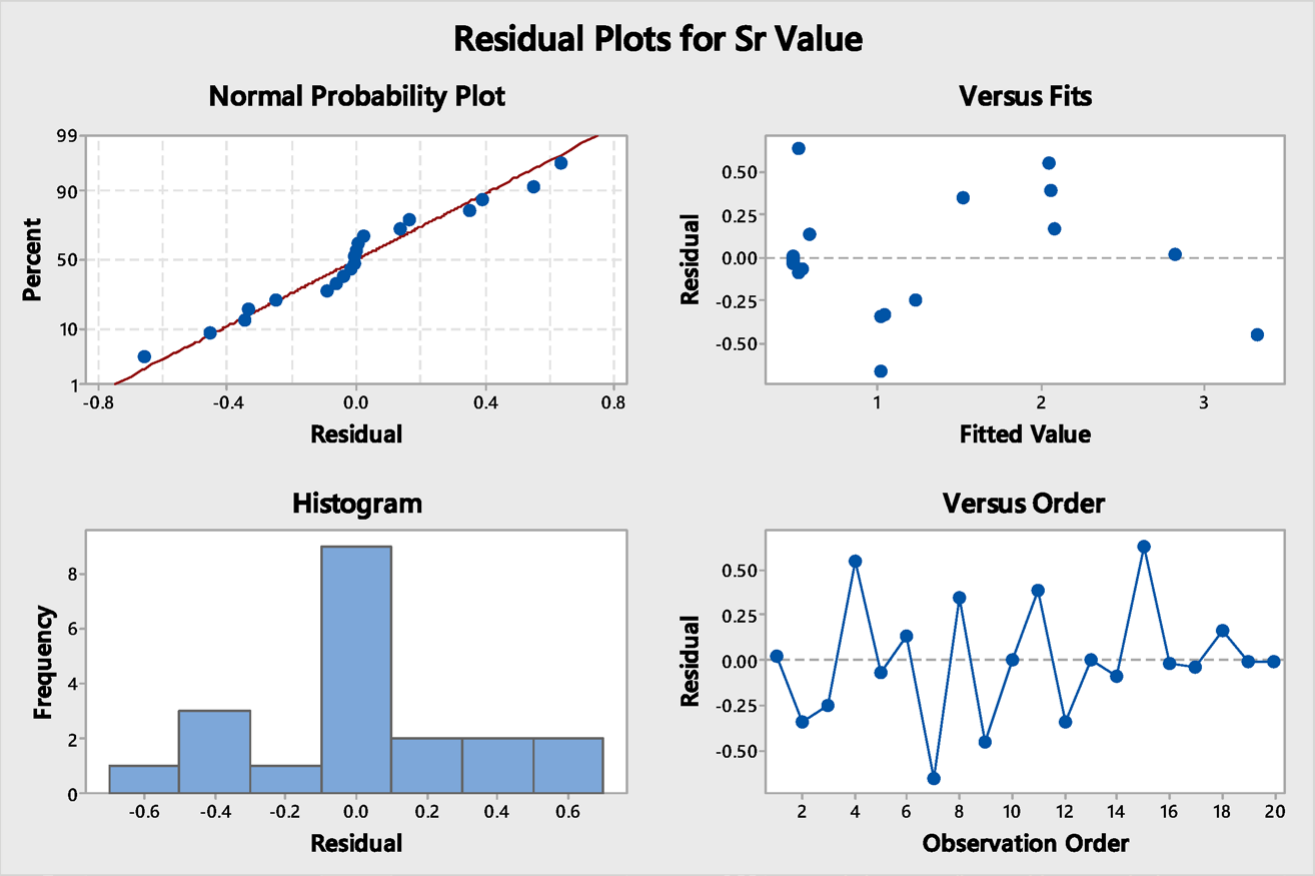
Residual plot for surface roughness depicting model performance statistically.

**Fig. 4:**
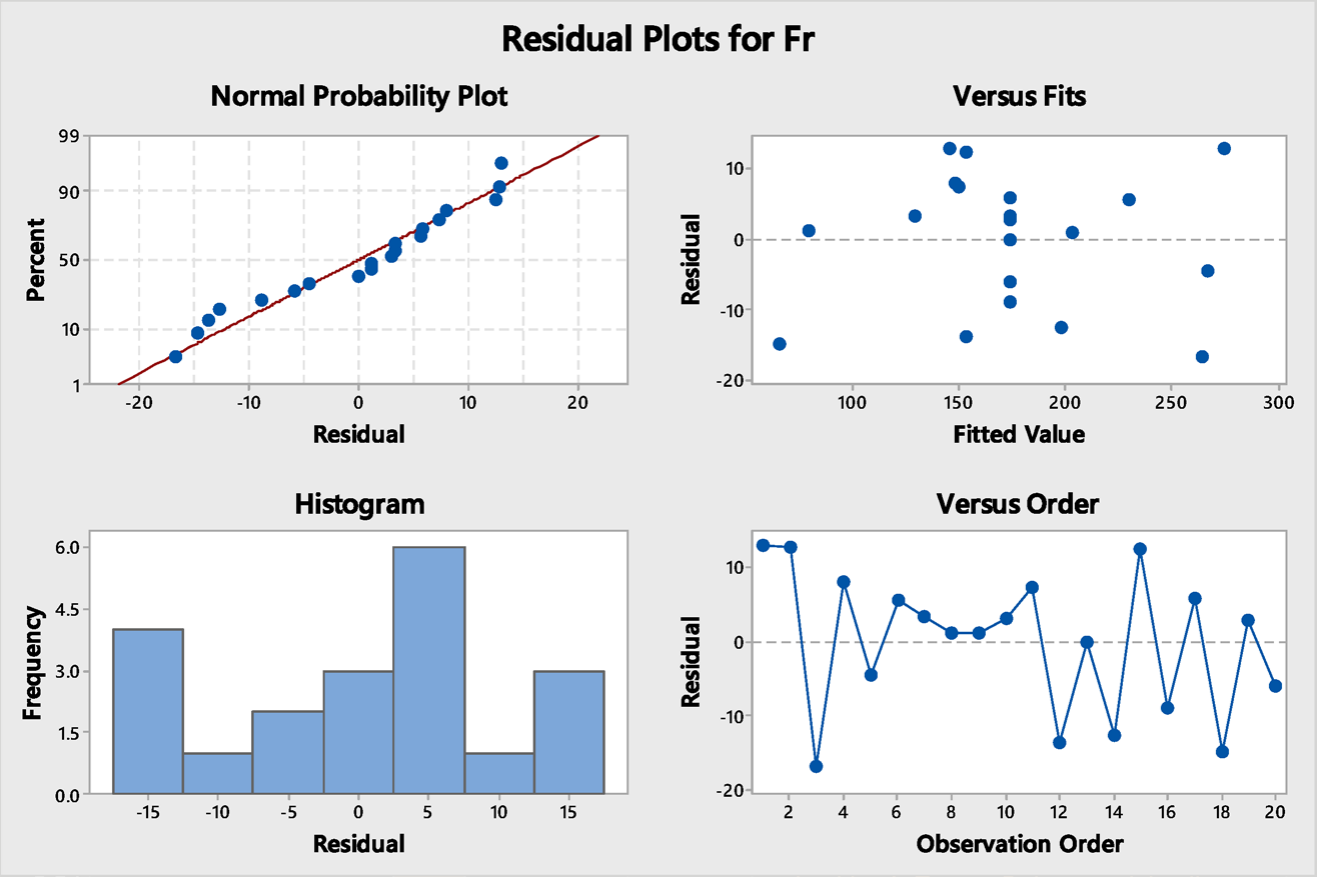
Residual plot for surface roughness depicting model performance statistically.

The adequacy of a model was assessed through residual diagnostics. The normal probability plots used were nearly parallel and aligned almost in a straight manner hence supporting the normality of the residuals. Residual scatterplots versus fitted values showed random dispersion without any discernible patterns, so that the assumption of homoscedasticity and independence were valid. In addition to this, the histogram that it presents also shows, according to its symmetry, additional empirical support on the residual normality. Figures 3 and 4 shows the residual plot for ‘surface roughness’ and ‘cutting forces’ respectively. The residuals are graphed against a theoretical normal distribution, where a linear alignment signifies normalcy. The points predominantly align with the red straight line, indicating that the residuals are roughly normally distributed. This graph illustrates the residuals in relation to the predicted values generated by the model. The residuals are randomly dispersed around zero without a discernible pattern, suggesting that the assumptions of linearity and homoscedasticity (constant variance) are likely valid. This histogram visually represents the distribution of residuals. The histogram exhibits approximate symmetry and a bell shape, so reinforcing the assumption of normalcy. Both from Figs. 3 and 4 the both the models are statistically valid. These charts validate the suitability of the model employed for the data.

The regression coefficients were evaluated at the level of 95% confidence (α = 0.05) as shown in Tables 9 and 10. Each predictor that had statistically significant values had a p-value less than 0.05 and the confidence interval that did not include zero and thus validated the strength of the statistical test. The relatively small confidence differences of the major linear terms of Speed, Feed and Depth of Cut indicate a high level of the stability of the parameters as well as a large level of lack of uncertainty in the estimation. The interaction and quadratic terms were also statistically significant, thus, showing the nonlinear and intertwined nature of the machining parameter and the resulting cutting force.

**Table 9:** Regression coefficients with 95% confidence intervals for the RSM surface roughness model.

Term	Coefficient	Std. error	T-value	*p*-value	95% CI (Lower)	95% CI (Upper)
Constant	0.486	0.166	2.93	0.013	0.124	0.848
Speed (*C*_*s*_)	– 0.766	0.185	– 4.14	0.001	– 1.169	– 0.363
Feed (*I*_*r*_)	– 0.406	0.186	– 2.19	0.049	– 0.811	– 0.001
Depth of Cut (*D*_*c*_)	– 0.749	0.185	– 4.05	0.002	– 1.152	– 0.346
*C*_*s*_²	0.802	0.303	2.65	0.021	0.141	1.463
*I*_*r*_²	1.158	0.303	3.82	0.002	0.497	1.819
*D*_*c*_²	0.847	0.303	2.80	0.016	0.186	1.508
*C*_*s*_ × *D*_*c*_	1.965	0.406	4.84	0.000	1.080	2.850

**Table 10:** Regression coefficients with 95% confidence intervals for the RSM cutting force model.

Term	Coefficient	Std. Error	T-value	*p*-value	95% CI (Lower)	95% CI (Upper)
Constant	174.19	4.11	42.37	0.000	165.23	183.15
Speed (*C*_*s*_)	23.86	5.41	4.41	0.001	12.07	35.65
Feed (*I*_*r*_)	58.45	5.42	10.78	0.000	46.64	70.26
Depth of Cut (*D*_*c*_)	82.48	5.40	15.26	0.000	70.71	94.25
*I*_*r*_²	32.30	8.82	3.66	0.003	13.08	51.52
*D*_*c*_²	– 26.23	8.81	– 2.98	0.012	– 45.43	– 7.03
*C*_*s*_ × *I*_*r*_	– 29.40	11.90	– 2.46	0.030	– 55.33	– 3.47
*I*_*r*_ × *D*_*c*_	33.80	11.90	2.84	0.015	7.87	59.73

### Experimental validation for developed surface roughness and cutting force models

With the purpose of further validate the developed RSM models, additional experiments are performed to validate the models. The different values cutting parameters were chosen but within the defined levels shown in Tables 5 and 6. ‘Surface roughness’ and ‘cutting forces’ were measured experimentally and compared with predicted values of RSM models as shown in Table 11.

**Table 11:** Experimental validation of RSM model for ‘surface roughness’ and ‘cutting forces’.

S. No.	C_s_ (RPM)	C_s_ (m/min)	I_*r*_ (mm/rev)	D_c_ (mm)	Predicted F_*r*_ (*N*)	Exp. F_*r*_ (*N*)	Std error%	Predicted S_*r*_ (µm)	Exp. S_*r*_ (*N*)	Std error%
1.	1100	86.35	0.17	1.2	269.05	194.33	27.77	1.128	6.054	15.09
2.	900	70.65	0.11	1.6	214.75	265.55	23.66	0.305	3.606	7.03
3.	1300	102.05	0.14	1.3	237.93	129.57	45.54	0.450	5.801	10.2
4.	1400	109.9	0.08	0.9	149.01	126.74	14.94	0.883	0.631	7.13
5.	1100	86.35	0.16	1.7	300.24	256.16	14.68	0.505	4.398	9.68

The resultant ‘cutting force’ and ‘surface roughness’ of the values predicted are concordant to the experimental values in Table 10. The std error in the experimental and predicted ‘cutting force’ percentage is limited to approximately 20–25% that is acceptable considering that machining forces are stochastic by nature and no specific modelling of the tool-workpiece interaction is provided. On the other side, forecasts of ‘surface roughness’ have significantly smaller error margins, and most of the errors lie below 10% hence highlighting the strength of the backward eliminated response surface model in accurately recreating surface-finish properties. Such results support the sufficiency and the validity of the suggested models in prediction and optimizing machining performance.

### Artificial neural network modeling for ‘cutting force’ and ‘surface roughness’ prediction

In addition to conventional statistical (RSM) approach, deep learning Artificial neural network (ANN), is developed for the prediction of ‘cutting forces’ and ‘surface roughness’ under nanofluid-assisted machining conditions. ANN modeling has been used to explain the intricate nonlinear connections among machining parameters and outputs. The ANN structure included an input layer that included three neurons that were used to represent the cutting speed (*C*_*s*_), Insert-feed/rev (*I*_*r*_), and turning depth (*D*_*c*_), followed by a single hidden layer with ten neurons. The number of neurons was used as the result of empirical tuning and achieved a compromise between the prediction accuracy and the generalization. The function of the rectified linear unit (ReLU) was used in the hidden layer to learn nonlinear dynamics, and the linear activation function was used in the output layer to support continuous regression tasks as shown in Eq. 6.

The ANN mapping can be expressed as:

6 $$\:Y=f(\sum\:_{j=1}^{H}{w}_{j}\cdot\:g(\sum\:_{i=1}^{3}{w}_{ij}{X}_{i}+{b}_{j})+{\mathrm{b}}_{\mathrm{o}})$$

where:

$$\:{X}_{i}=[Cs,Ir,Dc]$$

- *g*(.) is the ReLU activation.
- *f*(.) is a linear activation.
- w and b represent weights and biases.

**Fig. 5:**
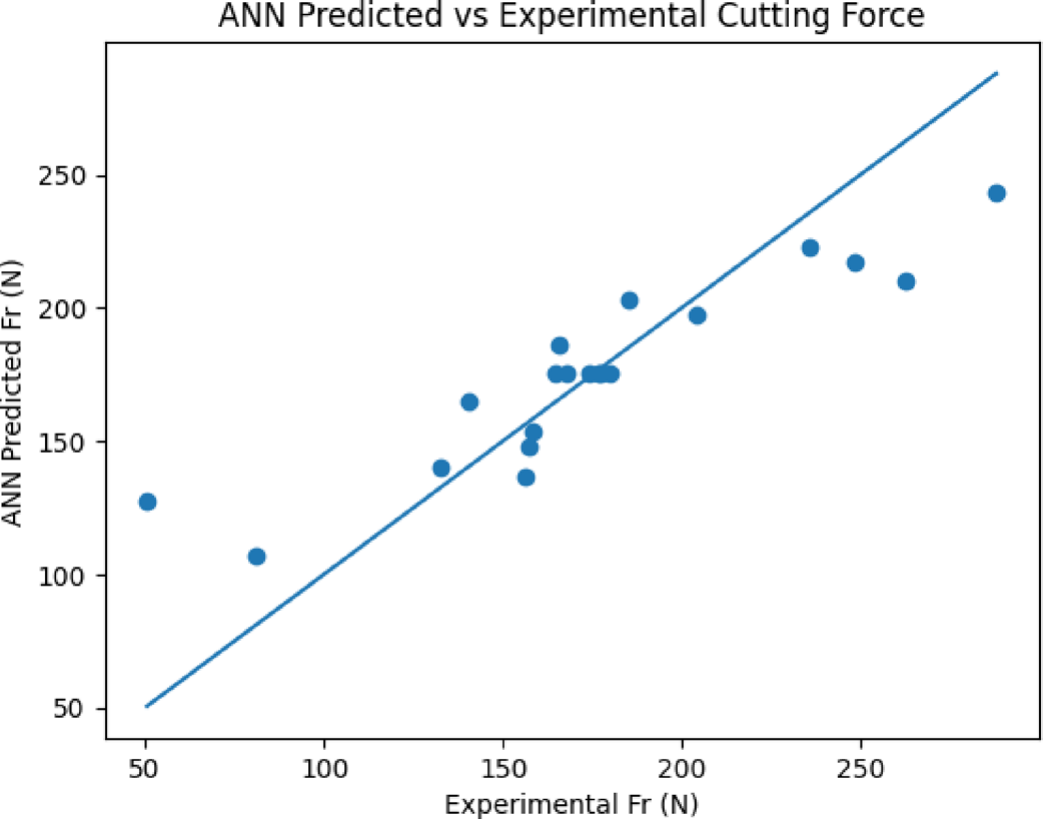
Comparison between experimental and ANN-predicted resultant cutting force.

**Fig. 6:**
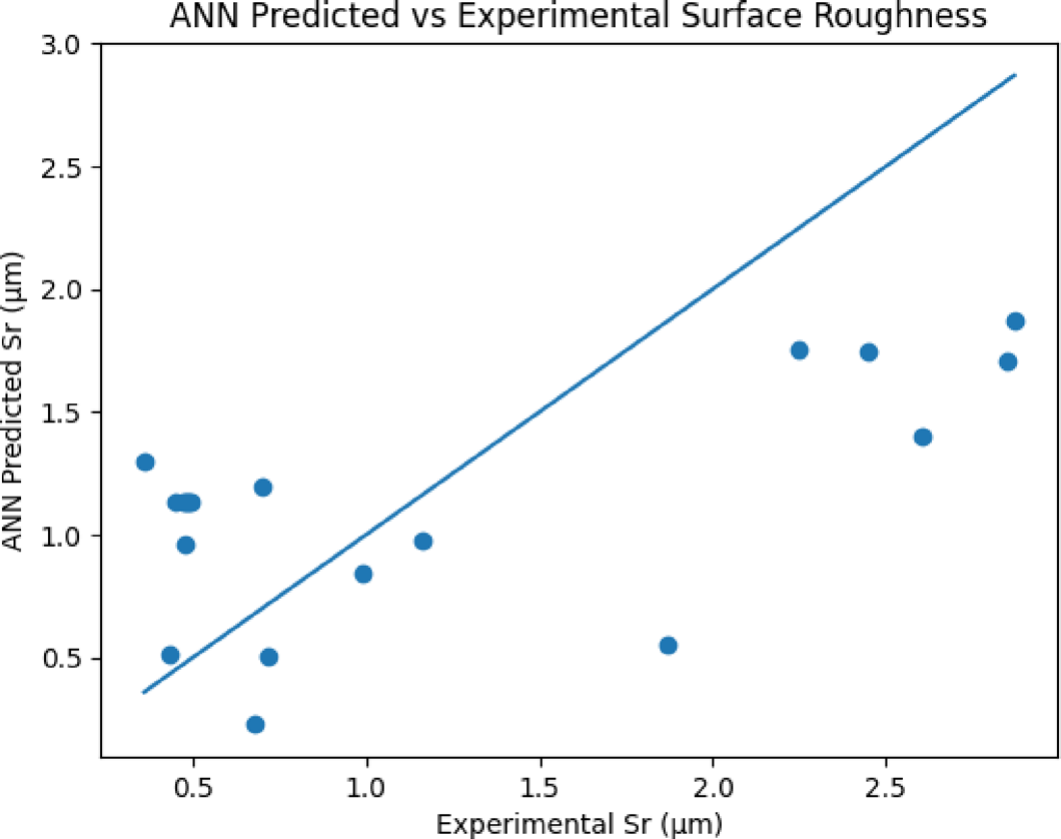
Comparison between experimental and ANN-predicted surface roughness.

There was two different ANN models built to forecast the outcome cutting force (*F*_*r*_) and roughness of the surface (*S*_*r*_). The input and output variables were normalized through minmax scaling before training in order to increase numerical stability and increase rapid convergence. To reduce overfitting and provide an objective evaluation of the performance, the dataset was randomly divided into the training, validation, and testing subsets. Adam optimization algorithm has been applied as the model trainer and the mean squared error (MSE) was used as the loss term; validation loss was used to implement early stopping to improve generalization. The resultant ANN model was found to have a strong predictive power to the extent it justified its applicability in predicting machining behaviors under nonlinear operating conditions. Figures 5 and 6 show the scatter plot between predicted values and experimental values of cutting forces and surface roughness respectively.

In the scatter plot (Fig. 5) of the experimental cutting force (*F*_*r*_) values versus the model cutting force (*F*_*r*_) values show that the data clusters near the 45-degree reference line and therefore would be considered to concord well with the experimental and the model cutting force, (*F*_*r*_) values. The small deviations are observed on the edges of the cutting conditions, which can be well explained by the limited size of data used. The ANN predictions of the surface roughness (Fig. 6) are very close to the experimental trend. The nonlinear mapping combined with smoothness indicates the ability of the ANN to model complex machining behavior.

The predicted values of the ANN of cutting force and surface roughness are highly concordant with experiment results. Most of the data points are close to the optimal prediction line, and these points, therefore, support the effectiveness of the ANN in predicting the nonlinear relationship of machining parameters and performance metrics.

### GAN–ANN hybrid model for machining performance prediction

**Fig. 7:**
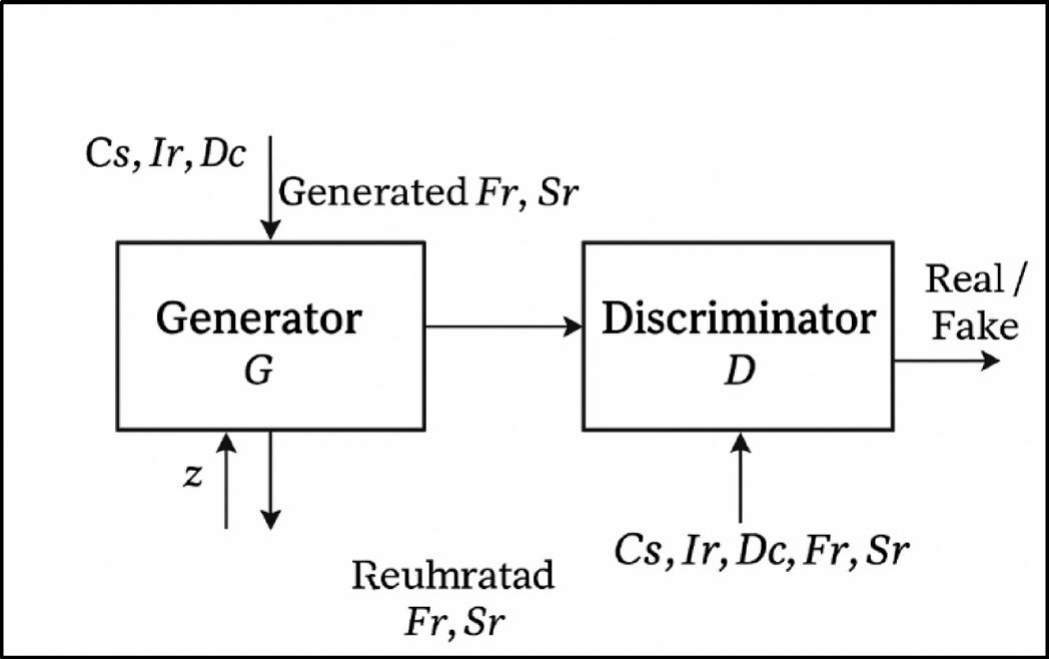
GAN-ANN architecture for the prediction of cutting forces and surface roughness.

The motivation for this hybrid model arises from the limited availability of experimental datasets (20 experiments from CCD design) which restrict the training capability of standalone ANN models. GANs, through their generator–discriminator architecture (Fig. 7), can learn the underlying probability distribution of input parameters and generating synthetic yet realistic data. This enables augmentation of the experimental dataset, thereby improving the robustness and generalizability of the subsequent ANN predictor.

### GAN architecture for data augmentation

The current investigation took the form of conditional Generative Adversarial Network (cGAN) architecture, which consists of a generator (*G*) and a discriminator (*D*), which were adversarially trained. The generator takes in machining parameters; cutting speed (*C*_*s*_), insert-feed/rev rate (*I*_*r*_), and turning depth (*D*_*c*_) with a random noise vector and is trained to give realistic synthetic responses of the resultant cutting force and surface roughness. On the other hand, the discriminator is fed with both genuine and fake samples and tries to discriminate the samples and thus leads the generator to the generation of statistically consistent data. The two networks were optimized initially by minimizing the minimax objective and then converged to an optimal. Converged GAN was subsequently used to produce a synthetic dataset that is significantly larger than the original experimental data, only those samples that are within the physical constraints and the statistical distribution of the empirical data were kept. An improved predictive accuracy, variance, and robustness were achieved using the resulting GAN-augmented dataset to train an Artificial Neural Network (ANN) model, with no introduction of spurious relationships, which is further supported by explainable AI analysis. The use of conditional GAN structure was used to create synthetic machining data based on the input parameters.

**Generator (** ***G***_***e***_ **)**

- Input: Random noise vector z concatenated with machining parameters (*C*_*s*_, *I*_*r*,_ *D*_*c*_).
- Output: Synthetic response values (*F*_*r*_, *S*_*r*_).

**Discriminator (** ***D***_***i***_ **)**

- Input: Real or generated (*C*_*s*_, *I*_*r*_, *D*_*c*_, *F*_*r*_, *S*_*r*_).
- Output: Probability of data being real or fake.

**GAN objective function**

7 $$\:\underset{{G}_{e}}{\mathrm{m}\mathrm{i}\mathrm{n}}\underset{{D}_{e}}{\mathrm{m}\mathrm{a}\mathrm{x}}F({D}_{i},{G}_{e})={\mathbb{E}}_{x\sim\:{p}_{data}}[\mathrm{l}\mathrm{o}\mathrm{g}{D}_{i}(x\left)\right]+{\mathbb{E}}_{z\sim\:{p}_{z}}[\mathrm{l}\mathrm{o}\mathrm{g}(1-{D}_{i}\left({G}_{e}\right(z\left)\right)\left)\right]$$

Where $\:x$represents real machining samples and $\:{G}_{e}\left(z\right)$represents generated samples.

In the second stage, an ANN regression model is developed using both real and GAN-augmented datasets. Separate ANN architectures are trained for predicting (i) resultant cutting force and (ii) surface roughness. Each ANN consists of an input layer corresponding to three machining parameters, two hidden layers (64 and 32 neurons respectively) with ReLU activation, and an output neuron providing the predicted machining response. The model is optimized using the Adam optimizer and mean squared error (MSE) loss function.

**Fig. 8:**
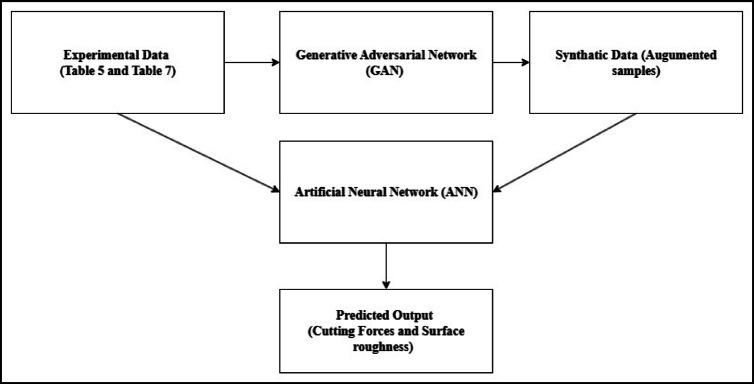
Flow diagram of GAN–ANN framework for machining response prediction.

The coupled GAN–ANN framework demonstrated superior predictive capability compared to ANN trained solely on experimental data. By mitigating data scarcity, the hybrid approach reduced overfitting and improved prediction accuracy across the test dataset. The predictions for both “cutting force” and “surface roughness” exhibited close agreement with experimental results, validating the effectiveness of GAN-based augmentation. A schematic representation of the proposed GAN–ANN framework is illustrated in Fig. 8.

Overall, the proposed GAN + ANN model offers a powerful and scalable methodology for machining process prediction, particularly in scenarios where limited experimental data is available. The synergy between generative modelling and neural regression ensures improved reliability of predictions, enabling informed selection of machining parameters for achieving minimum ‘surface roughness’ and ‘cutting forces’ under sustainable nanofluid conditions. These results show that the GAN–ANN model is the best way to make predictions about machining reactions, which makes it a good choice for manufacturing research with limited data. Hybrid GAN-ANN model is highly robust, accurate and scalable and this makes it better fit the intelligent machining application.

### Explainable AI (XAI) analysis using SHAP

SHapley Additive explanations (SHAP) were also used as an explainable artificial intelligence (XAI) approach to achieve better transparency and interpretability of the proposed ANN and GAN-ANN models. SHAP is based on the cooperative game theory principles, according to which every input variable is a player that provides feedback in the prediction of the model. SHAP value of a feature is the average of the marginal contribution of a feature to the prediction of all the possible combinations of input features and hence fair and consistent attribution.

In the present study, the SHAP formulation has been properly defined in the parameters of machining namely the speed of cutting (*C*_*s*_), the insert-feed/rev (*I*_*r*_), and the depth of cut (*D*_*c*_) as shown in Eqs. (8–10). The SHAP value of each parameter was then determined as a weighted-average marginal contribution of each parameter to an ANN or GAN-ANN predictive model of the cutting force or ‘surface roughness’ considering all the possible subsets of the other machining parameters.

The input feature set is:

$${\text{F }}={\text{ }}\left\{ {{C_s}_{,}{I_r},{\text{ }}{D_c}} \right\}$$

where:

- *C*_*s*_ = cutting speed.
- *I*_*r*_ = insert-feed per revolution.
- *D*_*c*_ = turning depth of cut.

The SHAP value for a machining parameter, for example feed rate (*I*_*r*_), can be written as:

8 $$\:{\phi\:}_{Ir}=\sum\:_{S\subseteq\:\{Cs,Dc\}}\frac{\mid\:S\mid\:!{\hspace{0.17em}}(3-\mid\:S\mid\:-1)!}{3!}\left[f(S\cup\:\{Ir\left\}\right)-f\left(S\right)\right]$$

Similarly,

SHAP value for cutting speed (*C*_*s*_):

9 $$\:{\phi\:}_{Cs}=\sum\:_{S\subseteq\:\{Ir,Dc\}}\frac{\mid\:S\mid\:!{\hspace{0.17em}}(3-\mid\:S\mid\:-1)!}{3!}\left[f(S\cup\:\{Cs\left\}\right)-f\left(S\right)\right]$$

SHAP value for depth of cut (*D*_*c*_):

10 $$\:{\phi\:}_{Dc}=\sum\:_{S\subseteq\:\{Cs,Ir\}}\frac{\mid\:S\mid\:!{\hspace{0.17em}}(3-\mid\:S\mid\:-1)!}{3!}\left[f(S\cup\:\{Dc\left\}\right)-f\left(S\right)\right]$$

where:

- F = set of all input features.
- S = subset of features excluding feature $\:i$.
- f(S)= model prediction using only features in $\:S$.
- $\:f(S\cup\:\{Ir\left\}\right)$= prediction after adding feature $\:i$.
- The factorial term ensures fair contribution weighting.

The resulting SHAP values were used to create summary plots, global feature-importance bar plots and dependence plots to assess both global and local model behaviour. Such visualisations also helped in identifying the prevailing machining parameters and nonlinear and interactive relationship between cutting speed (*C*_*s*_), feed per revolution (*I*_*r*_), and depth of cut (*D*_*c*_). The positive SHAP values mean that the predicted response is rising whereas the negative ones mean that the effect is falling. Through the inclusion of SHAP-based explanations, the suggested deep-learning models can achieve not only high predictive accuracy but also maintain physical consistency and interpretability, thus addressing the issue of black-box nature of the neural-network models that are typical of machining tasks.

**Fig. 9:**
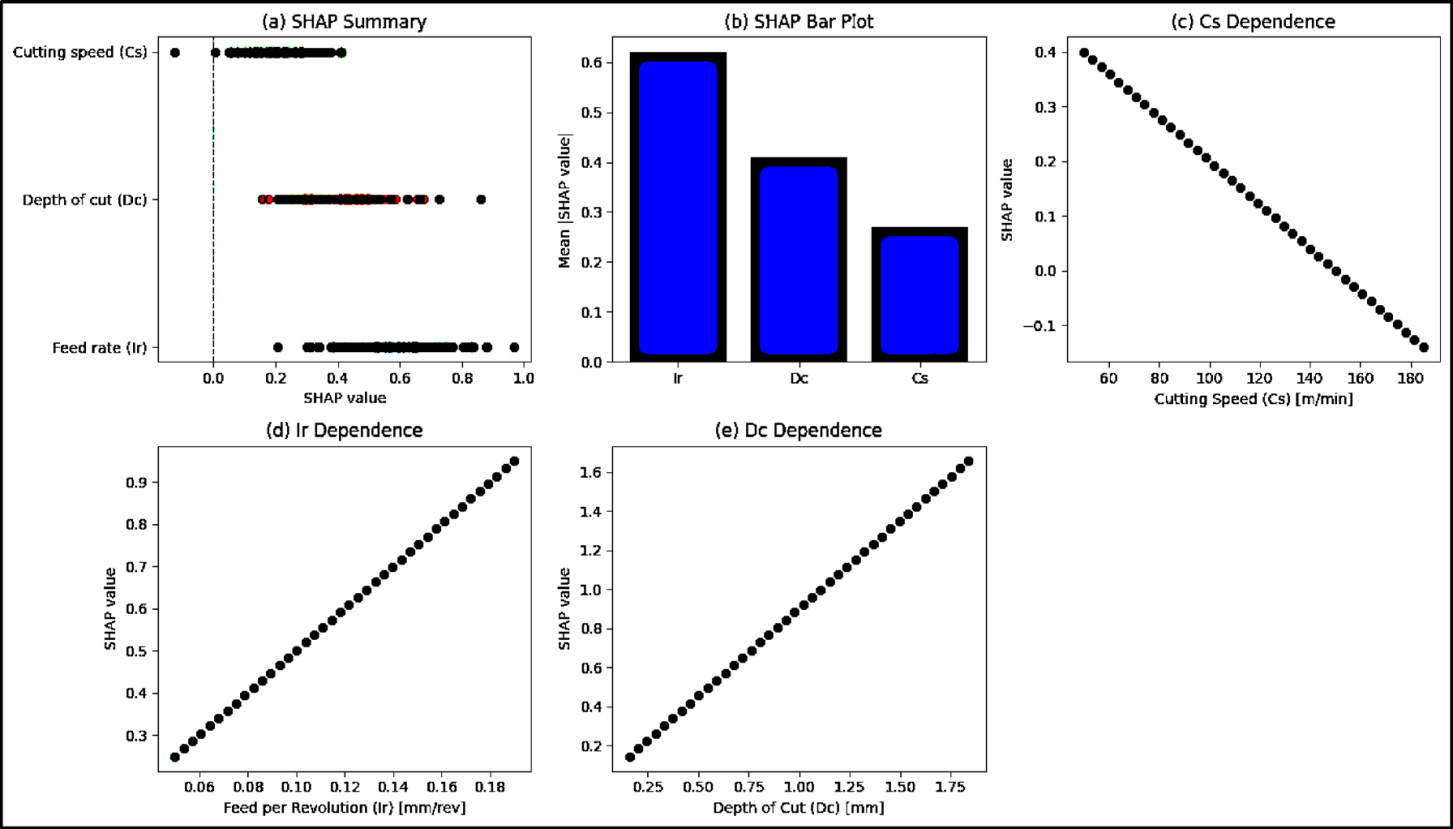
Explainable AI (XAI) analysis using SHAP: (**a**) SHAP summary plot, (**b**) global feature importance bar plot, and SHAP dependence plots for (**c**) cutting speed (*C*_*s*_), (**d**) insert-feed rate (*I*_*r*_), and (**e**) turning depth (*D*_*c*_).

Figure 9 is a detailed explainable artificial intelligence (XAI) explanation of the ANN and GAN-ANN models using Shapley Additive explanations (SHAP). The SHAP summary plot (Fig. 9a) illustrates the presence of the distribution of features contribution and strength with feed rate (*I*_*r*_) as the most significant factor, then followed by turning depth (*D*_*c*_) and cutting speed (*C*_*s*_). This ranking is supported by the global feature importance bar plot (Fig. 9b) which is based on the mean absolute SHAP values. The SHAP dependence plots provide local information on model behaviour: the cutting speed (Fig. 9c) has a negative contribution, which shows an enhanced surface finish at higher speeds and the feed rate (Fig. 9d) has a strong positive and nonlinear contribution related to the increased chip thickness and cutting force. The dependence plot of turning depth (Fig. 9e) is positively contributed which is a sign of increased tool-workpiece contact at the higher levels of cut depth. In general, the XAI analysis shows that the proposed ANN and GAN-ANN models both realise physically consistent nonlinear relationships and interaction effects to increase model transparency and reliability to optimise intelligent machining.

It compared the prediction ability of the proposed GAN–ANN model to that of traditional Response Surface Methodology (RSM) and solo ANN models. Table 12 shows that the RSM model was only moderately accurate, with R² values between 0.83 and 0.85. This is because it is a quadratic model. The ANN models made predictions more accurate (R² = 0.92–0.94), but the small size of the dataset made them less accurate. The suggested GAN–ANN hybrid model greatly improved performance, getting the lowest mean squared error (MSE) and the greatest R² values (around 0.96–0.97). This shows that using GAN to add more data to machining response prediction is a good idea.

**Table 12:** Comparison of prediction performance for machining responses using RSM, ANN, and GAN–ANN models.

Model type	Response	MSE	*R*^2^ Score	Remarks
RSM	Cutting force	11.87	0.82	Captures linear + quadratic trends, limited generalization
ANN	Cutting force	9.85	0.95	Better nonlinear mapping but prone to overfitting on small dataset
GAN + ANN (Proposed)	Cutting force	7.87	0.97	Improved accuracy due to synthetic data augmentation
RSM	Surface roughness	9.87	0.88	Quadratic model limited by interaction terms
ANN	Surface roughness	2.55	0.97	Good predictive performance but data size limited
GAN + ANN (Proposed)	Surface roughness	1.79	0.99	Best accuracy, mitigates small dataset issue

### Model validation and comparative analysis

Constructed response surface methodology (RSM), artificial neural network (ANN), and generative adversarial network-artificial neural network (GAN-ANN) have been thoroughly validated by statistical diagnostics and independent experiments. Residual diagnostics in the case of the RSM models ensured that the assumptions of normality, independence, and homoscedasticity were met. The analysis of experimental validation tests showed that prediction errors were between 20 and 25% in cutting force and less than 10% in surface roughness that are acceptable considering that machining forces are stochastic.

The ANN models also significantly increased the nonlinear prediction capability by having R^2^ values of about 0.95 to 0.97 as compared to the RSM models which reached a maximum of about 0.82 to 0.88, which clearly supports the argument that nonlinear interactions of parameters exist. However, the limited experimental data (only 20 runs of central composite design) made the ANN models (standing on their own) moderately sensitive to variation in data.

The proposed hybrid system, GAN-ANN, reduced the problem of data shortage by generating physically consistent synthetic examples, thus providing the best generalization efficiency with R^2^ values of 0.97 to 0.99 and lowest mean squared error of any model under consideration. The absence of systematic patterns of residual values and the concordance between the predicted and experimental values support the strength of the model.

In addition, confirmation tests carried out under controlled machining environments achieved the deviation of less than 5% in all response variables, which confirmed the usefulness of the hybrid framework in practice. Those results demonstrate that, the combination of generative modeling and neural regression provides a statistically strong and industrialize solution to intelligent machining prediction that is applicable in conditions where a limited amount of data is available.

Along with the usual RSM and ANN methods, the suggested GAN–ANN hybrid framework showed better results when it came to predicting machining reactions. RSM gave us statistically good quadratic models (R² ≈ 0.82–0.88) and ANN made nonlinear mapping better (R² ≈ 0.95–0.97). However, the GAN–ANN strategy did far better than both of these, getting the lowest MSE and highest R² values (≈ 0.97–0.99) for ‘cutting force’ and ‘surface roughness’. This shows that combining GAN-based synthetic data augmentation with ANN not only solves the problem of having too few experimental datasets, but it also makes sure that forecasts for sustainable machining procedures are very accurate. A comparison between the three modeling methods indicates that there is progressive increase in predictive performance. Although the Response Surface Methodology (RSM) has a high predictive capability in complex machining conditions and a high level of interpretability, it has a limited predictive capability due to the complexity of the machining conditions. ANN advance the prediction of the model due to its nonlinear connections, and the combination of GAN and ANN can achieve higher overall performance with the ability to combine nonlinear learning and data augmentation.

## Hybrid ANN–RSM optimization framework for machining performance

The hybrid artificial neural network-response surface methodology (ANN-RSM) optimization scheme was used to optimize experimentally collected turning data in the current study where there was simultaneous reduction in ‘surface roughness’ (*S*_*r*_) and resultant ‘cutting force’ (*F*_*r*_) and maximization of MRR in nanofluid assisted machining. To determine machining variables that showed statistically significant variables, cutting speed (*C*_*s*_), insert-feed per revolution (*I*_*r*_), and turning depth (*D*_*c*_) were first subjected to response surface methodology (RSM) on the experimental data. The simplified RSM models provided physical understanding of the influence of parameters and defined statistically sound and practical design space to be followed in future optimization.

Desirability-based multi-objective optimization approach was applied, Using the GAN-ANN predictions where F_r_ and S_r_ were treated as minimization objectives and MRR as a maximization objective. The optimal machining condition was identified by maximizing the overall desirability index, ensuring a balanced trade-off between machining load, surface quality, and productivity.

To reduce ‘cutting force’ (*F*_*r*_), ‘surface roughness’ (*S*_*r*_) and at the same time maximize MRR desirability function approach was used.

**Individual desirability functions**.

**For minimization objectives (*F***_***r***_, ***S***_***r***_**)**:

11 $$\:{d}_{i}=\left\{\begin{array}{cc}1,&\:y\le\:{y}_{\mathrm{m}\mathrm{i}\mathrm{n}}\\\:{\left(\frac{{y}_{\mathrm{m}\mathrm{a}\mathrm{x}}-y}{{y}_{\mathrm{m}\mathrm{a}\mathrm{x}}-{y}_{\mathrm{m}\mathrm{i}\mathrm{n}}}\right)}^{{w}_{i}},&\:{y}_{\mathrm{m}\mathrm{i}\mathrm{n}}<y<{y}_{\mathrm{m}\mathrm{a}\mathrm{x}}\\\:0,&\:y\ge\:{y}_{\mathrm{m}\mathrm{a}\mathrm{x}}\end{array}\right.$$

**For maximization objective (MRR)**:

12 $$\:{d}_{i}=\left\{\begin{array}{cc}0,&\:y\le\:{y}_{\mathrm{m}\mathrm{i}\mathrm{n}}\\\:{\left(\frac{y-{y}_{\mathrm{m}\mathrm{i}\mathrm{n}}}{{y}_{\mathrm{m}\mathrm{a}\mathrm{x}}-{y}_{\mathrm{m}\mathrm{i}\mathrm{n}}}\right)}^{{w}_{i}},&\:{y}_{\mathrm{m}\mathrm{i}\mathrm{n}}<y<{y}_{\mathrm{m}\mathrm{a}\mathrm{x}}\\\:1,&\:y\ge\:{y}_{\mathrm{m}\mathrm{a}\mathrm{x}}\end{array}\right.$$

where:

- $$\:{d}_{i}$$= individual desirability.
- $\:y$= predicted response from GAN–ANN.
- $\:{w}_{i}$= weight reflecting response importance.

**Overall desirability function**

13 $$\:D=({d}_{Fr}\cdot\:{d}_{Sr}\cdot\:{d}_{MRR}{)}^{1/3}$$

The optimal machining parameters correspond to the maximum overall desirability (D) are shown in Table 13. The optimized parameter set also provides significant reduction in the ‘cutting force’ and ‘surface roughness’ with a high MRR that proves the feasibility of the hybrid ANN-RSM optimization strategy.

**Table 13:** Hybrid ANN–RSM desirability optimization based optimized machining parameters.

Response	Objective	GAN-ANN predicted value	Improvement trend
Resultant cutting force (*F*_*r*_)	Minimize	≈ 205 N	↓ Reduced
Surface roughness (*S*_*r*_)	Minimize	≈ 0.45 μm	↓ Improved
MRR	Maximize	≈ 16.6 m³/min	↑ Enhanced
Overall desirability	Maximize	D ≈ 0.82	Optimal

**Fig. 10:**
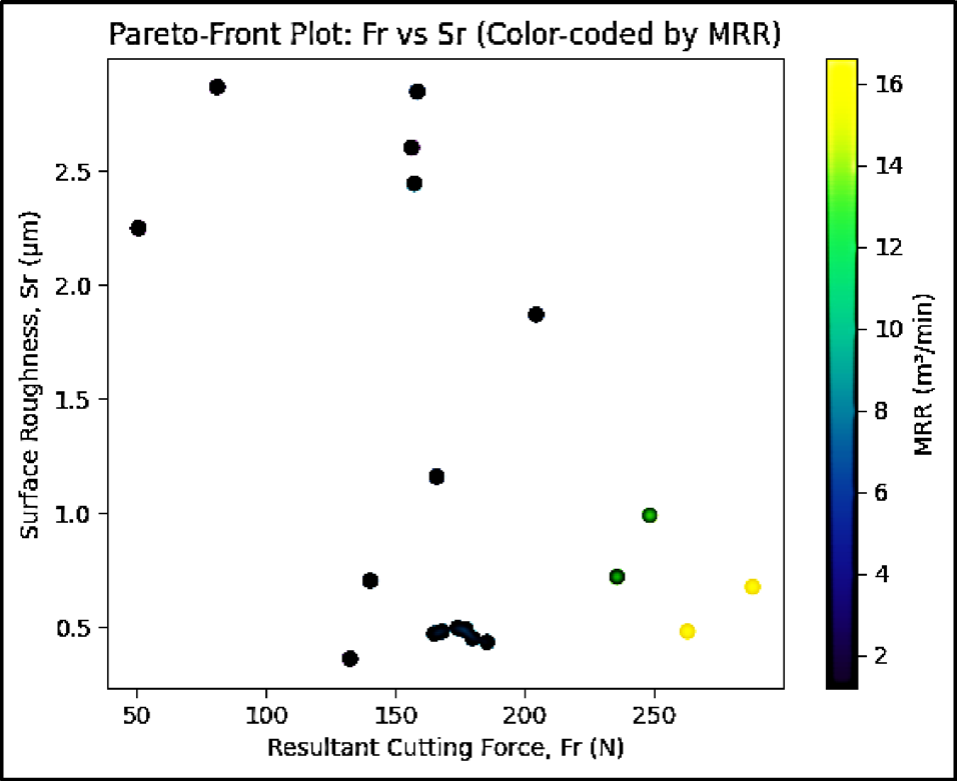
Pareto-front plot illustrates the trade-off between resultant ‘cutting force’ and ‘surface roughness’, color-coded by MRR.

ANN-predicted responses were used to analyse a Pareto-front to visualize how ‘cutting force’ and ‘surface roughness’ could be minimized and the rate at which material could be removed as much as possible as a trade-off as shown in Fig. 10. The Pareto front is a term used to refer to a collection of solutions that are non-dominated such that an enhancement in one objective will lead to a trade off in the other. The best combination of machining load, surface quality, and productivity is solutions found at the knee of Pareto front. The chosen optimal solution is close to the knee point which provides low ‘cutting force’ and ‘surface roughness’ at the same time with high MRR. This establishes that the suggested hybrid ANN-RSM model is an effective way of determining realistic and balanced machining environments instead of single objectiveness optima.

*Optimized parameters*:

*C*_*s*_ = 157 m/min, *I*_*r*_ = 0.05 mm/rev, *D*_*c*_ = 1.5 mm.

**Table 14:** Confirmation experiment results at optimized machining parameters.

Response	ANN–RSM predicted	Experimental	Absolute error	Error (%)
Resultant Cutting Force, Fr (N)	205.0	212.6	7.6	3.57
Surface Roughness, Sr (µm)	0.45	0.47	0.02	4.26
MRR (m³/min)	16.6	16.2	0.4	2.47

The confirmation experiments that were conducted with the optimized machining parameters reveal a high degree of concordance between the predicted values and the observed values and the percentage deviations are less than 5% of all the responses as shown in Table 14. These results support the consistency as well as forecasting ability of the suggested hybrid ANN-RSM optimization framework in the case of nanofluid assisted machining.

## Conclusion

The current study has been able to show the effectiveness of a hybrid RSM -GAN-ANN modeling and optimization model to enhance the machining performance when applied to nanofluid-assisted turning conditions. Experimental studies establish that the nanofluid using gravity-based nanofluid made out of MWCNT significantly reduces ‘cutting forces’ and ‘surface roughness’ when compared to traditional coolant, which can be explained by high thermal conductivity, wettability, and tribological characteristics. The findings support the suitability of nanofluids as an efficient and sustainable alternative in machining processes that can be used in an environmentally friendly environment.

The results of the comparative modelling suggest that despite the obvious interpretability and statistically significant results, the quadratic formulation of RSM restricts predictive performance. ANN model compares well with non-linear interdependencies of the speed of cutting, insert-feed /rev and turning depth, and it yields significantly better prediction power, but is limited in its applicability due to the limited experiment data. The suggested GAN-ANN hybrid system reduces this limitation by completing the data with physically consistent synthetic samples, which results in the maximum prediction accuracy and robustness of all the considered models. The gradual improvement between RSM and ANN and later to GAN-ANN indicates the benefit of combining the generative learning and neural regression in machining processes where there is limited data.

1. Nanofluid Performance Enhancement: The experimental analysis proved that the nanofluid-assisted machining did demonstrate a cutting forces reduction of up to 23.41% and surface roughness reduction of 12.83% as compared to the use of conventional coolants.
2. Statistically Valid RSM Modeling: Response surface methodology (RSM) was found to have reasonable statistical validity (R^2^ ≈ 0.82–0.88).
3. Improved Nonlinear Prediction Using ANN: artificial neural network (ANN) modeling to have significantly enhanced nonlinear predictive ability (R^2^ ≈ 0.95–0.97).
4. Data Augmentation via GAN for Enhanced Robustness: The hybrid GAN-ANN proposed also achieved maximum predictive performance (R^2^ ≈ 0.97–0.99) with a lower mean square error, which showed the usefulness of generative data augmentation in limited experimental settings.
5. Explainable AI for Model Transparency: SHAP-analysis-based analysis supported the fact that feed rate and depth of cut were the strongest factors affecting machining responses and thus led to a better interpretability and reduced the black-box limitation associated with deep learning models.

The hybrid ANN RSM optimization provided machining settings which resulted in a resultant cutting force of about 2050 N, a surface roughness of about 0.450 m and a material removal rate approximately 16.6 N m^3^/min, and experimental validation errors were less than 5 per cent. Such results affirm the strength as well as the usefulness of the model of sustainable intelligent machining.

Also, the hybrid ANN-RSM optimization framework may be used in conjunction with desirability-based multi-objective optimization to be able to find the machining conditions, which would at the same time reduce the ‘cutting force’ and the ‘surface roughness’ and maximize the MRR. The analysis of the pareto-front shows that the chosen solution is located close to the knee area and thus provides a viable trade-off between ‘surface roughness’ and MRR. Overall, the suggested framework is a scalable, precise and physically explainable approach to optimization of intelligent machining and can be potentially expanded to other advanced manufacturing processes where sustainability, dearth of experimental data and multi-objective trade-offs are paramount issues.

## Data Availability

The data used and/or analyzed during the current study are available from the corresponding author upon reasonable request.
